# Supplementary material for: Temperature modulates stress response in mainstream anammox reactors
Source: Commun Biol. 2021 Jan 4;4:23. doi: 10.1038/s42003-020-01534-8 (PMC7782526; doi:10.1038/s42003-020-01534-8)
Supplement: Supplementary file 2 — Supplementary Information [file 42003_2020_1534_MOESM2_ESM.pdf]

Supplementary Information

Temperature modulates stress response in anammox reactors

Robert Niederdorfer[1], Damian Hausherr[2], Alejandro Palomo[3], Jing Wei[4], Paul Magyar[5], Barth Smets[3], Adriano Joss[2], Helmut Bürgmann[1]

[1]Eawag, Swiss Federal Institute for Aquatic Science and Technology, Department of Surface Waters-Research and Management, 6047 Kastanienbaum, Switzerland,

[2]Eawag, Swiss Federal Institute for Aquatic Science and Technology, Department of Process Engineering, 8600 Duebendorf, Switzerland

[3]Department of Environmental Engineering, Technical University of Denmark, Kgs Lyngby, Denmark

[4]Empa, Swiss Federal Laboratories for Materials Science and Technology, Laboratory for Air Pollution & Environmental Technology, 8600 Dübendorf, Switzerland

[5]Department of Environmental Sciences, University of Basel, Basel, Switzerland

This file includes:

Supplementary figures 1-8
Supplementary tables 1-2

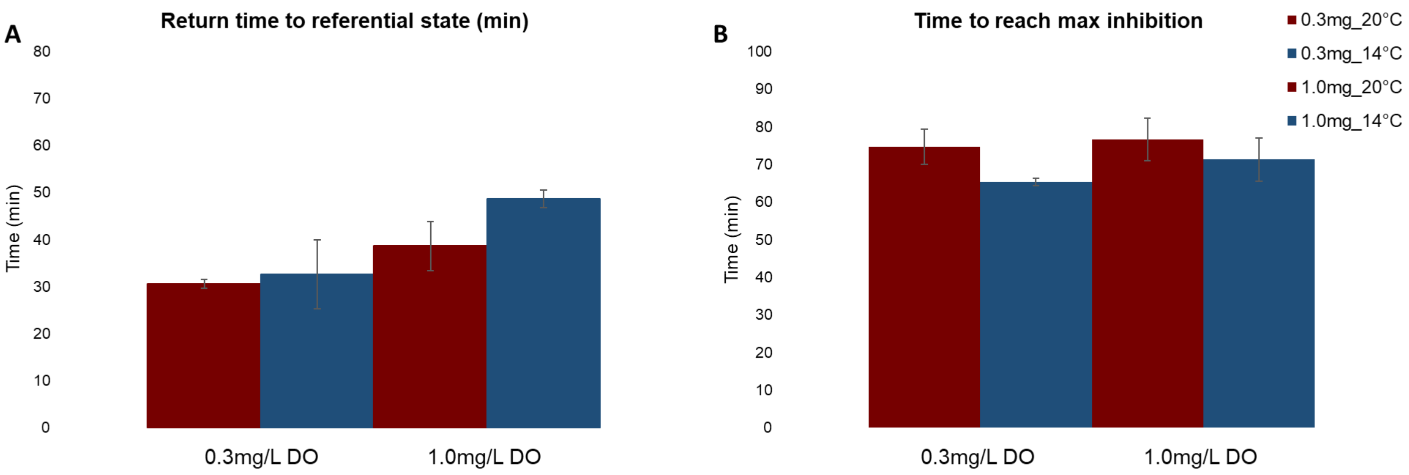

Supplementary Figure 1 A Return time to baseline performance levels (NH_4^+ removal rates) in minutes after dissolved oxygen perturbations under different temperature regimes (20 °C, red; 14 °C, blue). Left bars denote 0.3 mg L^{-1} DO stress response while right bars reflect the 1.0 mg L^{-1} DO stress response. **B** Time in minutes to reach maximum impact of the applied DO disturbance. Triplicate reactors were averaged for this graph.

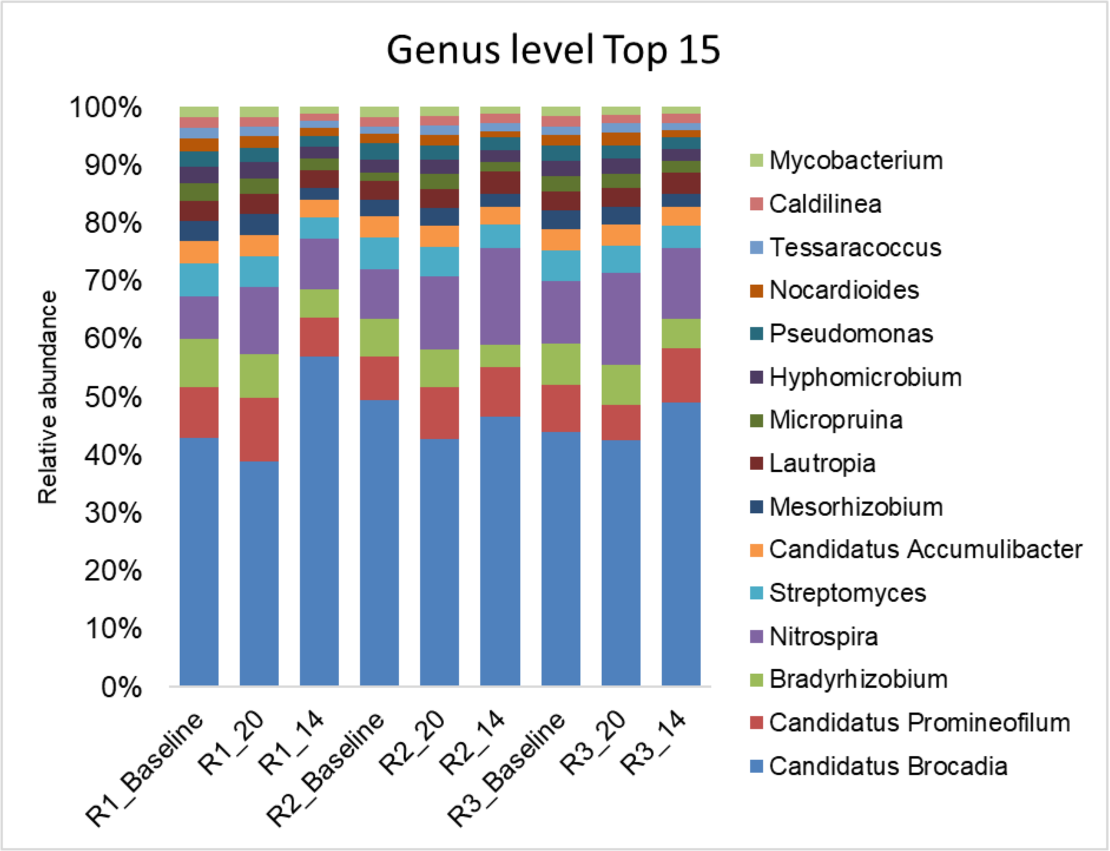

Supplementary Figure 2 Relative abundance of the top 15 bacterial genera of the biofilm carrier community derived from metagenomic sequencing. Labels denote Reactor (R1, R2, R3) and time of sampling, **_Baseline** (Pooled from 20 °C and 14 °C experiment), **_20** (after the 20 °C experiment), **_14** (after the 14 °C experiment). Colors represent the different bacterial

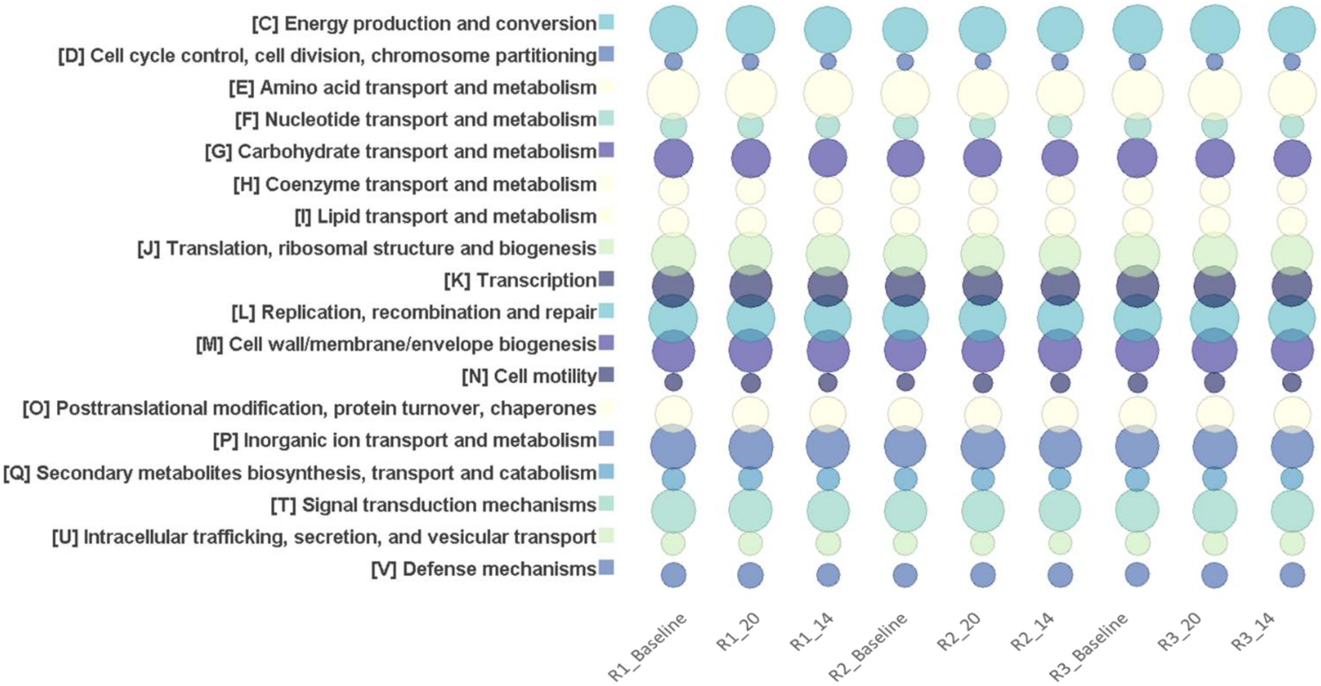

Supplementary Figure 3 Gene abundances (GPM) categorized into **C**luster of **O**rthologues **G**roups (COG). Labels denote Reactor (R1, R2, R3) and time of sampling, **_Baseline** (Pooled from 20 °C and 14 °C experiment), **_20** (after the 20 °C experiment), **_14** (after the 14 °C experiment). Size of bubbles correspond to the relative abundance of the group.

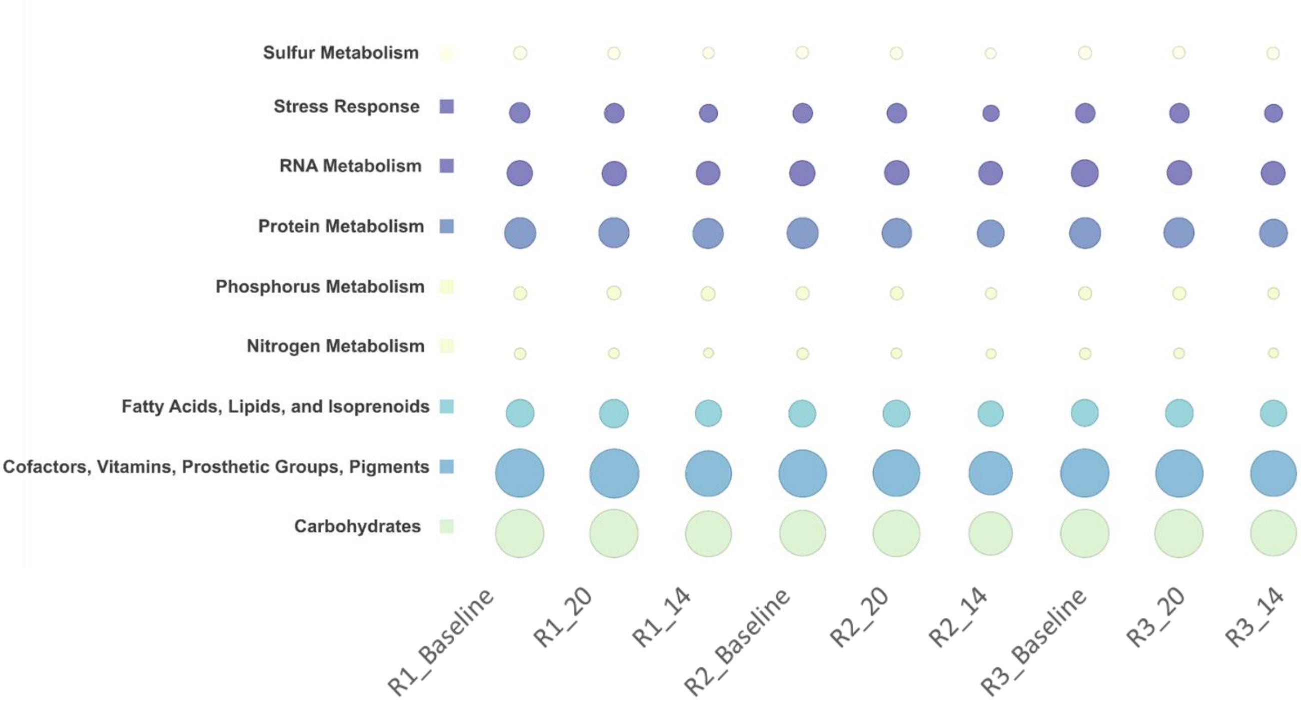

Supplementary Figure 4 Gene abundances categorized into **Seed subsystems**. Labels denote Reactor (R1, R2, R3) and time of sampling, **_Baseline** (Pooled from 20 °C and 14 °C experiment), **_20** (after the 20 °C experiment), **_14** (after the 14 °C experiment). Size of bubbles correspond to the relative abundance of the system.

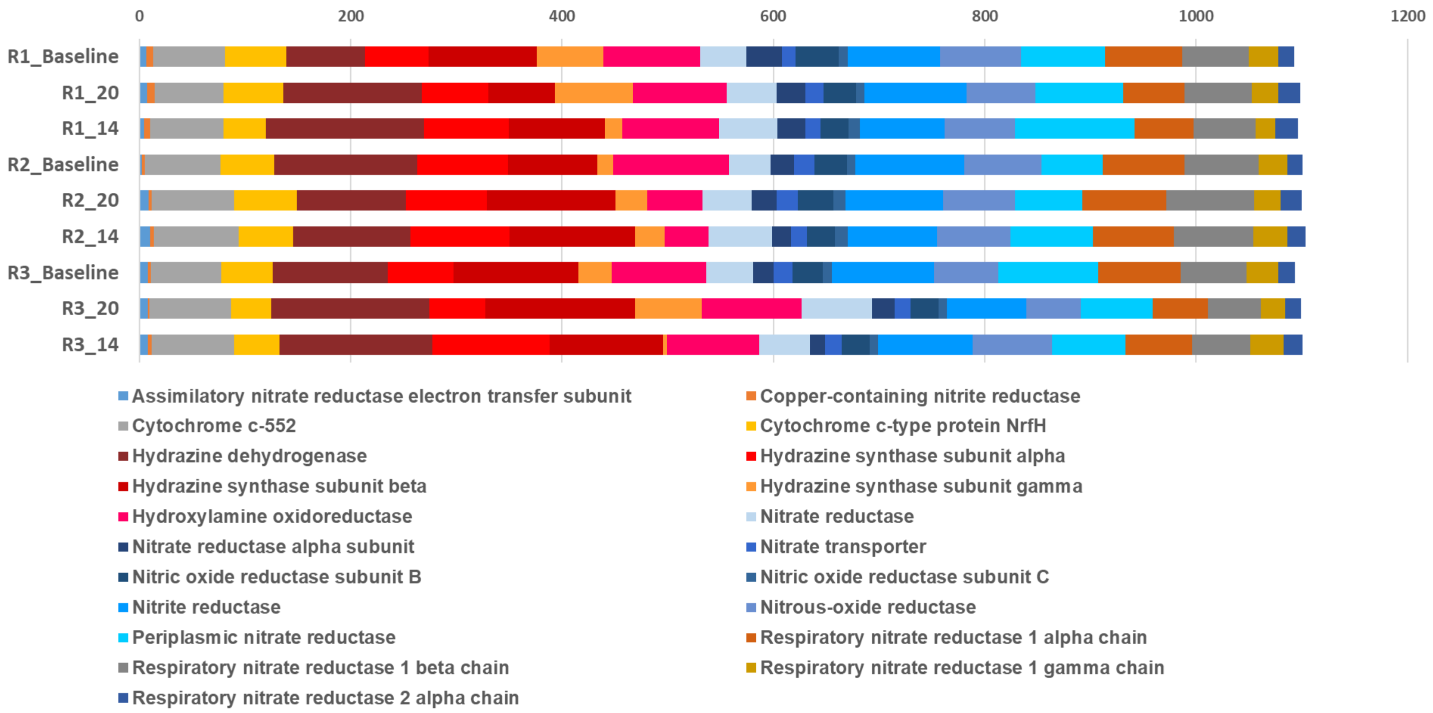

Supplementary Figure 5 Abundances of the most prominent genes from the nitrogen cycle expressed as genes per million. Reddish colours correspond to genes involved in the anammox cycle, blueish to the denitrification pathway. Labels denote Reactor (R1, R2, R3) and time of sampling, **_Baseline** (Pooled from 20 °C and 14 °C experiment), **_20** (after the 20 °C experiment), **_14** (after the 14 °C experiment).

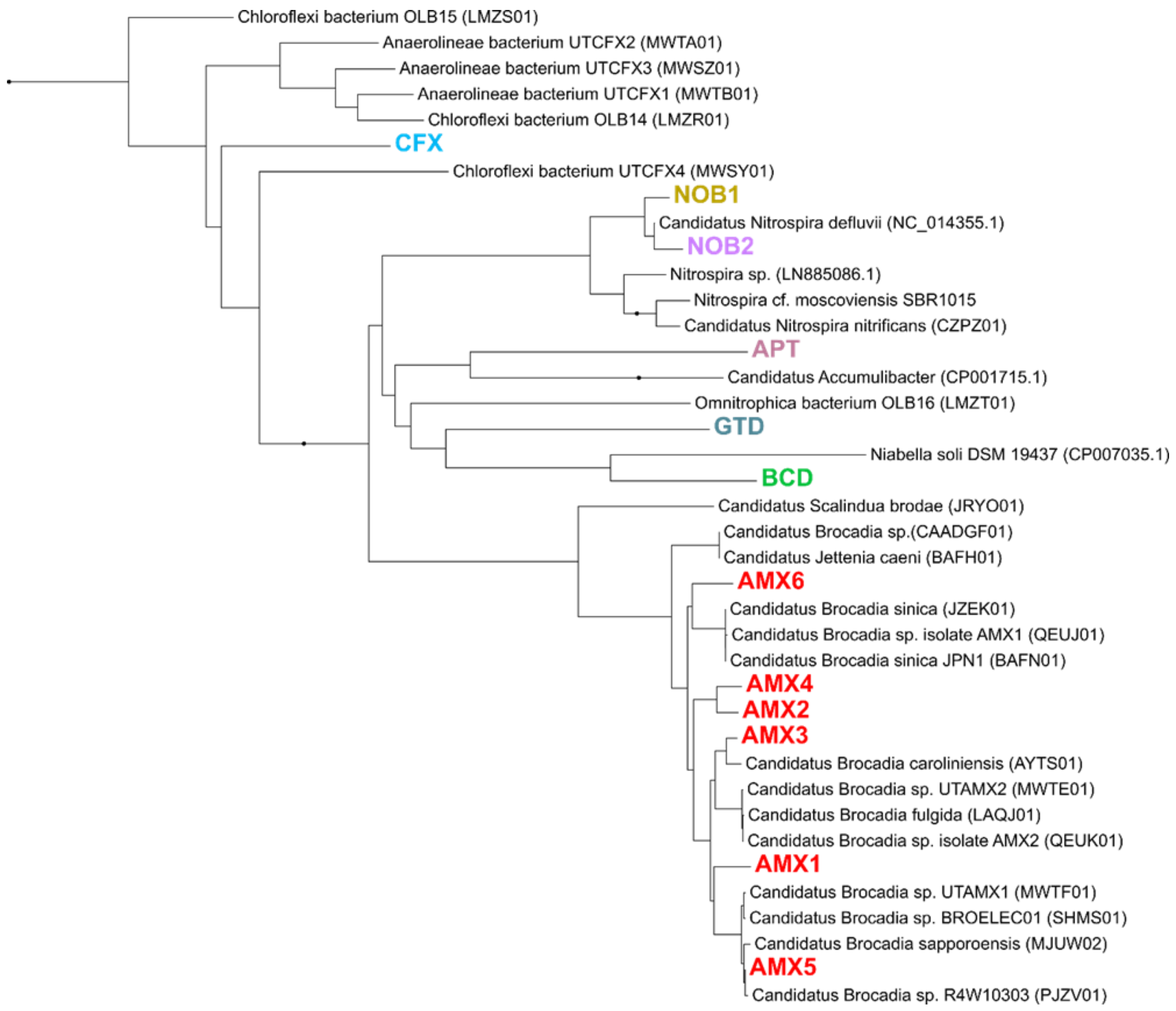

Supplementary Figure 6 Phylogenetic tree of all recovered draft genomes from the anammox bioreactor. Tree includes MAGs recovered from this study (different colours) and closely related genomes downloaded from the NCBI genome repository. GenBank accession numbers for each genome are provided in parentheses. The tree was constructed using RAxML based on a set of 37 concatenated universal single-copy marker genes.

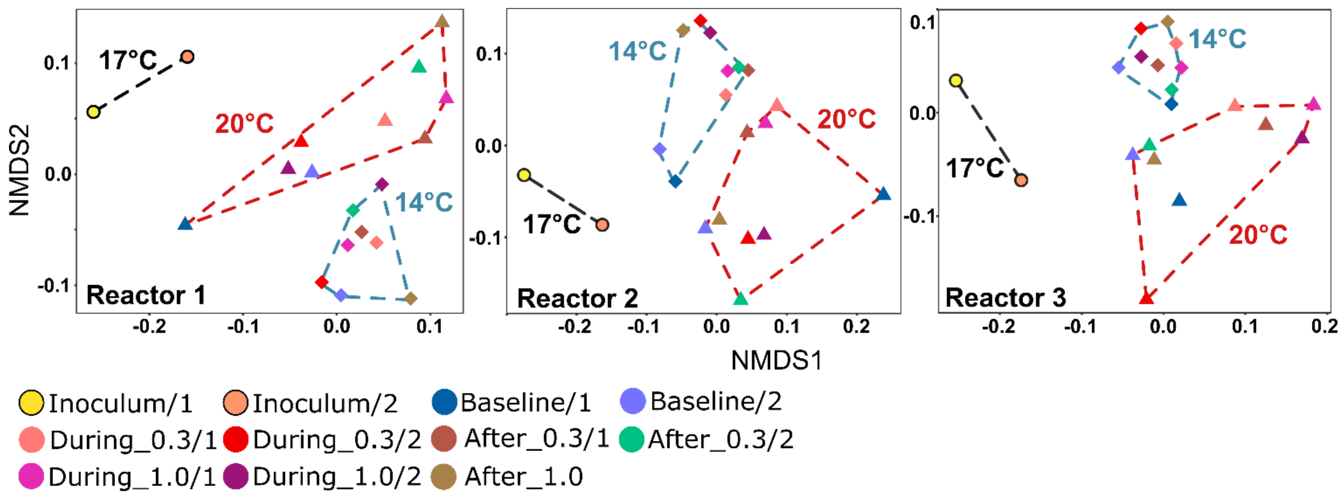

Supplementary Figure 7 nMDS analysis based on Jaccard dissimilarities depicting overall gene expression during the time-points of the experiment under different temperatures for all reactors. Colors of clusters denote the respective temperature of the experiment (black: Inoculum, red:20 °C, blue:14 °C). Reactor1 is missing one Baseline sample of the 14 °C experiment due to insufficient coverage of the metatranscriptome. Stress values: R1:0.12; R2:0.13; R3:0.12

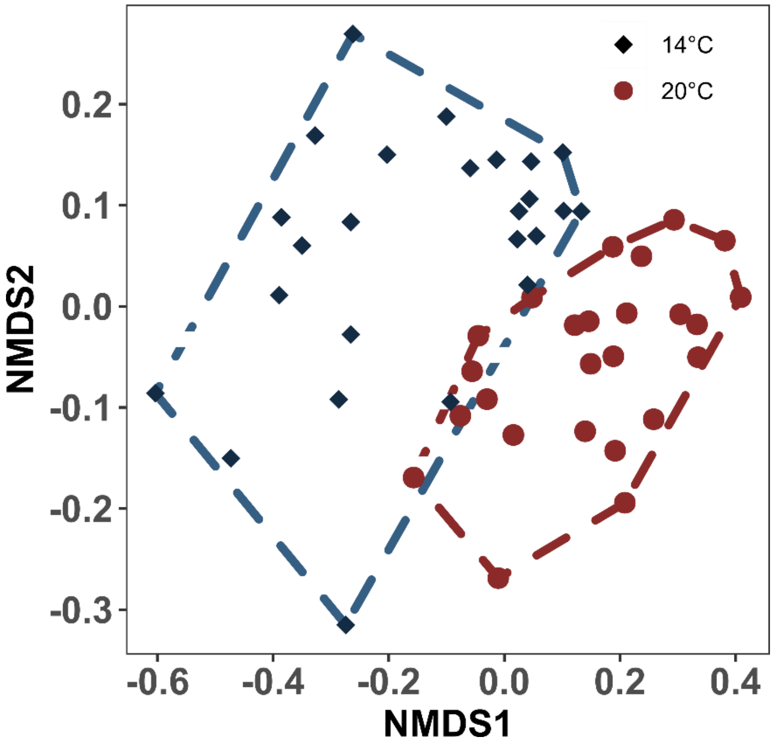

Supplementary Figure 8 nMDS based on Jaccard dissimilarity depicting all genes involved in the Nitrogen cycle. Each dot represents a timepoint. The colour denotes the temperature regime. Hulls highlight also the temperature regime. Stress: 0.115

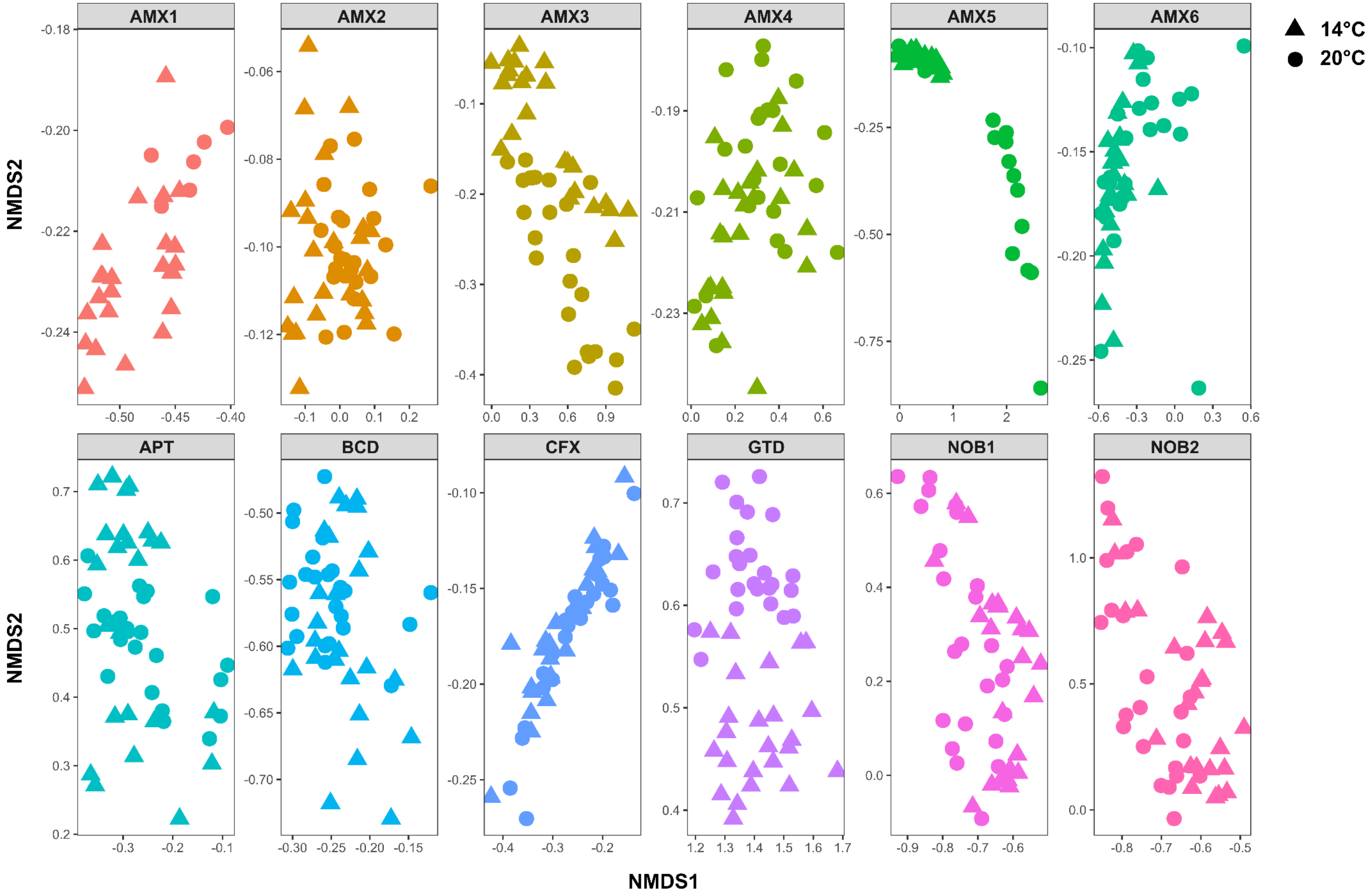

Supplementary Figure 9 nMDS (Jaccard dissimilarity) based on the COG expression profile for each MAG. Colors denote different MAGs shapes correspond to the temperatures. Each dot represents the COG profile of the corresponding MAG on a respective time-point. Symbols denote the temperature (dot 20 °C; triangle 14 °C). Stress: 0.074

Supplementary Table 1 Nitrogen cycle genes presence/absence in metagenome assembled genomes. Numbers correspond to the amount of gene copies found within the respective genome. For multi-gene subsystems (e.g. *narGHI*) we summed up the copies from each individual gene.

		AMX1	AMX2	AMX3	AMX4	AMX5	AMX6	CFX	NOB1	NOB2	APT	GTD	BCD
**Anammox**	***hao***	**1**	**6**	**3**	**6**	**4**	**7**	**x**	**x**	**x**	**x**	**x**	**x**
	hzs	**1**	**2**	**1**	**x**	**x**	**2**	**x**	**x**	**x**	**x**	**x**	**x**
	hdh	**3**	**4**	**3**	**4**	**3**	**4**	**x**	**x**	**x**	**x**	**x**	**x**
	nirK	**x**	**x**	**1**	**x**	**x**	**x**	**x**	**x**	**x**	**x**	**x**	**x**
**Denitrification**	***narGHI***	**x**	**x**	**1**	**x**	**x**	**x**	**x**	**x**	**x**	**x**	**2**	**1**
	napAB	**x**	**x**	**x**	**x**	**x**	**x**	**x**	**x**	**x**	**2**	**x**	**x**
	nirK	**x**	**x**	**x**	**x**	**x**	**x**	**x**	**1**	**1**	**1**	**x**	**1**
	nirS	**x**	**x**	**x**	**x**	**x**	**x**	**1**	**x**	**x**	**1**	**x**	**2**
	norBC	**1**	**1**	**1**	**1**	**1**	**1**	**x**	**x**	**x**	**x**	**x**	**2**
	nosZ	**x**	**x**	**x**	**x**	**x**	**x**	**1**	**x**	**x**	**1**	**1**	**1**
**DNRA**	***nrfAH***	**1**	**3**	**x**	**3**	**3**	**1**	**1**	**1**	**1**	**x**	**x**	**x**
**Assimimilatory nitrate reduction**	***narB***	**1**	**1**	**1**	**x**	**x**	**1**	**x**	**2**	**1**	**x**	**x**	**x**
	nasABC	**2**	**1**	**1**	**1**	**1**	**1**	**x**	**1**	**1**	**x**	**x**	**x**

Supplementary Table 2 Relative Abundance of MAGs and gene expression estimates, based on transcripts per million values of transcripts that mapped to each MAG

MAG	abundance	average mRNA count 20°C	average mapped mRNA (%) 20°C	average mRNA count 14°C	average mapped mRNA (%) 14°C	Transcript abundance (TPM) 20°C	Transcript abundance (TPM) 14°C
AMX1	13.70	2777994.89	17.68	3660796.41	17.02	59.23	57.02
AMX2	3.94	1432948.93	9.12	1480315.48	6.88	28.86	21.78
AMX3	1.13	143038.71	0.91	251727.00	1.17	2.92	3.75
AMX4	0.69	140002.86	0.89	204590.44	0.95	3.07	3.28
AMX5	0.46	95637.39	0.61	289596.33	1.35	2.14	4.73
AMX6	0.44	86592.11	0.55	130320.19	0.61	1.64	1.80
CLX	1.96	191590.18	1.22	185097.85	0.86	1.50	2.13
NOB1	1.07	86092.29	0.55	89529.78	0.42	1.43	1.09
NOB2	1.03	80927.39	0.52	91434.11	0.43	1.79	1.47
APT	0.74	16753.71	0.11	14252.93	0.07	0.18	0.11
GTD	0.48	51423.43	0.33	61076.19	0.28	0.81	0.70
BCD	0.47	22484.96	0.14	23642.22	0.11	0.39	0.30
