# Supplementary material for: Temperature modulates stress response in mainstream anammox reactors
Source: Commun Biol. 2021 Jan 4;4:23. doi: 10.1038/s42003-020-01534-8 (PMC7782526; doi:10.1038/s42003-020-01534-8)
Supplement: Supplementary file 3 — Description of Additional Supplementary Files [file 42003_2020_1534_MOESM3_ESM.pdf]

Description of Additional Supplementary Files

File Name: Supplementary Data 1
Description: Supplementary Data 1 contains the average Ammonia concentrations per cycle and half-hour resolution to produce Figure 1 and 2

File Name: Supplementary Data 2
Description: Supplementary Data 2 contains the raw gene transcript abundance table and all other datasets to produce Figure 3-7
